# Septal contributions to olfactory bulb interneuron diversity in the embryonic mouse telencephalon: role of the homeobox gene *Gsx2*

**DOI:** 10.1186/s13064-017-0090-5

**Published:** 2017-08-16

**Authors:** Shenyue Qin, Stephanie M. Ware, Ronald R. Waclaw, Kenneth Campbell

**Affiliations:** 1 Divisions of Developmental Biology, Cincinnati Children’s Hospital Medical Center, University of Cincinnati College of Medicine, Cincinnati, OH 45229 USA; 2 Molecular and Developmental Biology Graduate Program, University of Cincinnati College of Medicine, Cincinnati, OH 45229 USA; 3 Neurosurgery, Cincinnati Children’s Hospital Medical Center, University of Cincinnati College of Medicine, Cincinnati, OH 45229 USA; 4 Experimental Hematology and Cancer Biology, Cincinnati Children’s Hospital Medical Center, University of Cincinnati College of Medicine, Cincinnati, OH 45229 USA; 5 Department of Pediatrics and Medical and Molecular Genetics, Indiana University School of Medicine, Indianapolis, IN 46202 USA

**Keywords:** Neurogenesis, Neuronal specification, Olfactory bulb, Septum, Transcription factor

## Abstract

**Background:**

Olfactory bulb (OB) interneurons are known to represent diverse neuronal subtypes, which are thought to originate from a number of telencephalic regions including the embryonic dorsal lateral ganglionic eminence (dLGE) and septum. These cells migrate rostrally toward the OB, where they then radially migrate to populate different OB layers including the granule cell layer (GCL) and the outer glomerular layer (GL). Although previous studies have attempted to investigate regional contributions to OB interneuron diversity, few genetic tools have been used to address this question at embryonic time points when the earliest populations are specified.

**Methods:**

In this study, we utilized *Zic3-lacZ* and *Gsx2e-CIE* transgenic mice as genetic fate-mapping tools to study OB interneuron contributions derived from septum and LGE, respectively. Moreover, to address the regional (i.e. septal) requirements of the homeobox gene *Gsx2* for OB interneuron diversity, we conditionally inactivated *Gsx2* in the septum, leaving it largely intact in the dLGE, by recombining the *Gsx2* floxed allele using *Olig2* ^*Cre/+*^ mice.

**Results:**

Our fate mapping studies demonstrated that the dLGE and septum gave rise to OB interneuron subtypes differently. Notably, the embryonic septum was found to give rise largely to the calretinin^+^ (CR^+^) GL subtype, while the dLGE was more diverse, generating all major GL subpopulations as well as many GCL interneurons. Moreover, *Gsx2* conditional mutants (cKOs), with septum but not dLGE recombination, showed impaired generation of CR^+^ interneurons within the OB GL. These *Gsx2* cKOs exhibited reduced proliferation within the septal subventricular zone (SVZ), which correlated well with the reduced number of CR^+^ interneurons observed.

**Conclusions:**

Our findings indicate that the septum and LGE contribute differently to OB interneuron diversity. While the dLGE provides a wide range of OB interneuron subtypes, the septum is more restricted in its contribution to the CR^+^ subtype. *Gsx2* is required in septal progenitors for the correct expansion of SVZ progenitors specified toward the CR^+^ subtype. Finally, the septum has been suggested to be the exclusive source of CR^+^ interneurons in postnatal studies. Our results here demonstrate that dLGE progenitors in the embryo also contribute to this OB neuronal subtype.

**Electronic supplementary material:**

The online version of this article (doi:10.1186/s13064-017-0090-5) contains supplementary material, which is available to authorized users.

## Background

Olfactory bulb (OB) interneurons represent a highly diverse neuronal population that serve as important components in the relay of olfactory signals from the environment to the brain [1]. They are largely inhibitory and modulate local projection neuron activity by releasing gamma-aminobutyric acid (GABA) [2, 3]. The complex functions of OB interneurons are accomplished by their high diversity, which, at least in part, can be recognized as subtypes based on distinct biochemical markers being expressed [3, 4]. In addition, OB interneurons occupy distinct layers of the OB, allowing them to exert their functions through building connections selectively with tufted cells or mitral cells, the major projection neurons in the OB [5, 6, 7]. Interestingly, OB interneurons of different subtypes show varied preferences in layer localization and neuronal connectivity [3]. For example, tyrosine hydroxylase-labeled (TH^+^) dopaminergic interneurons and calbindin^+^ (CB^+^) interneurons are enriched in the glomerular layer (GL), whereas calretinin^+^ (CR^+^) interneurons are found in both GL and granule cell layer (GCL). The specific roles of the diverse OB interneuron subtypes in olfactory circuits is not well defined, however, studies have shown these interneurons originate from the embryonic ventral telencephalon and regional progenitor domains in the postnatal SVZ [2, 8, 9, 10].

Unlike the locally born projection neurons [11, 12, 13], OB interneurons are generated caudal to the bulb within the ventral telencephalon, largely the lateral ganglionic eminence (LGE) and septum, from embryonic day 12 (E12) until birth and subsequently from the postnatal and adult SVZ, which represents the derivative of these embryonic germinal zones [8, 14, 15, 16]. The newly specified neuroblasts migrate tangentially along the rostral migratory stream (RMS) to the OB, where they radially migrate to populate different layers and undergo maturation [17, 18]. Recently, it has been suggested that OB interneurons of distinct subtypes are produced by progenitor cells in different topological domains of the perinatal telencephalon [2, 7, 9, 10, 16]. For example, TH^+^ interneurons were shown to be generated from the LGE, whereas CR^+^ interneurons are suggested to be predominantly produced by the septum [9, 10, 16]. Despite the relatively detailed studies of the origins of OB interneuron subtypes, few genetic approaches have been taken to address the contributions of different progenitor domains to OB interneuron diversity at embryonic stages.

The normal generation and specification of OB interneurons are regulated by a number of transcription factors [7, 19]. For example, previous studies suggested that zinc finger transcription factor Sp8, which is expressed by many post-mitotic neuroblasts from both LGE and septum, is essential for the normal generation of CR^+^ and parvalbumin^+^ OB interneurons [20, 21]. Another zinc finger transcription factor Tshz1 is required for the normal generation of CB^+^ interneurons as well as the radial migration of multiple subtypes after neuroblasts ultimately reach the OB [22]. Finally, the TH^+^ subpopulation requires both Pax6 and Er81 (Etv1) for their normal generation [2, 23, 24, 25, 26]. While these transcriptional regulators are expressed in the migrating and differentiating neuroblasts, the homeobox gene *Gsx2* is highly expressed by progenitor cells in the LGE and septum, and has been shown to be critical for the normal generation of many OB interneuron subtypes [20, 27, 28, 29, 30, 31, 32]. Specifically, it was demonstrated that the generation of OB interneurons is severely compromised when Gsx2 is absent in the dorsal LGE (dLGE) [31]. Gsx2 is also highly enriched in the VZ progenitor cells of the septum, which represents another important source of OB interneurons at perinatal time points [2, 7, 9, 10, 14, 33]. Gsx2 is required for normal gene expression in the embryonic septum, including its downstream effector *Ascl1* and related targets [34]. However, the function of Gsx2 in the specification of septum-derived OB interneurons has not been examined.

In this study, we utilized two genetic fate-mapping tools to investigate the LGE and septal contributions to OB interneuron diversity. In addition, by using a conditional knockout strategy, we examined the role of *Gsx2* in the generation of septum-derived OB interneurons. Our data demonstrate that the LGE and septum give rise to OB interneuron subtypes differently, with the LGE being heterogeneous and the septum providing rather specifically the CR^+^ interneurons of the GL. Additionally, we show that *Gsx2* is required for the expansion of specified septal SVZ progenitors that give rise to CR^+^ interneurons.

## Methods

### Animals

*Olig2* ^*Cre/+*^ mice [35] and *Gsx2e-CIE* mice [36] were genotyped with the following primers: JaxCre-5′ (5′-GCGGTCTGGCAGTAAAAACTATC-3′) and JaxCre-3′ (5′-CCATGAGTGAACGAACCTGG-3′). *Gsx2* ^*flox/+*^, *Gsx2* ^*RA/+*^ and *Gsx2* ^*EGFP/+*^ alleles were genotyped as previously described [31, 32]. *Rosa* ^*tdTomato*^ (*Ai14*) mice were genotyped with the following primers: Rosa-tdTomato-5′ (5′-GGCATTAAAGCAGCGTATCC-3′) and Rosa-tdTomato-3′ (5′-CTGTTCCTGTACGGCATGG-3′) [37]. *Zic3-lacZ* BAC transgenic mice [38] were genotyped with the following primers: βgal5’ (5′-TGGGGAATGAATCAGGCCACGG-3′) and βgal3’ (5′-GCGTGGGCGTATTCGCCAAGGA-3′). The *Gsx1* knockout mice [39] and staged embryos were genotyped with the following primers: Gsx1-WT1 (5′-CGGGTGAAGCACAAGAAAGAAG-3′), Gsx1-WT2 (5′-CCAATGGTCCTCTAAAAGGCG-3′), Gsx1-MT1 (5′-GGTTCATCATCACTAATCACGACG-3′) and Gsx1-MT2 (5′-CGCTGTTCTCCCTCTTCCTCATCTC-3′).

For embryonic analysis, the morning of the vaginal plug observed was designated embryonic day (E)0.5. Embryos were fixed in 4% PFA overnight at 4 °C, extensively rinsed in PBS and cryoprotected in 30% sucrose in PBS. Embryos were embedded in Neg-50 embedding medium for frozen tissue sectioning (Thermo Scientific) and coronal or horizontal sections were obtained at 12 μm on a cryostat. Sections were mounted onto SuperFrost Plus Microscope Slides (Fisher Scientific) and stored at −20 °C until processed. Postnatal brains were collected at P14. Brains were removed from skull and fixed in 4% PFA overnight at 4 °C before being extensively rinsed in PBS and cryoprotected in 20% sucrose in PBS. Brains were then embedded in Neg-50 (Thermo Scientific) and coronal sections were obtained at 14 μm on a cryostat. Again sections were mounted onto SuperFrost Plus Microscope Slides (Fisher Scientific) and stored at −20 °C until staining.

### Immunohistochemistry

Primary antibodies were used at the following concentrations: rabbit anti-βgal, 1:1000 (Biogenesis); goat anti-βgal, 1:1000 (Biogenesis); chicken anti-βgal, 1:500 (Abcam); rabbit anti-calbindin, 1:2500 (a gift from Dr. Piers Emson, Babraham Institute); goat anti-calretinin, 1:2000 (Millipore); rabbit anti-Gsx2, 1:5000 [40]; rabbit anti-Ki67, 1:1000 (Abcam); rabbit anti-Mef2c, 1:2000 (Protein Tech Group); mouse anti-Neurofilament (NF-M), 1:200 (deposited to the Developmental Studies Hybridoma Bank by T.M. Jessell and J. Dodd); rabbit anti-Pax6, 1:1000 (Biolegend); goat anti-Sp8, 1:8000 (Santa Cruz Biotechnology); chicken anti-TH, 1:500 (Aves Labs), rabbit anti-panZic, 1:2000 (a gift from Dr. Stephen Brown, University of Vermont). Bright-field staining was obtained by using diaminobenzidine (DAB) as the chromogen following 2-h incubation in biotinylated goat anti-guinea pig (1:200, Vector Laboratories), horse anti-goat (1:200, Jackson Immunoresearch) or swine anti-rabbit (1:200, DAKO) and 1-h incubation in ABC solution (Vector Laboratories). Secondary antibodies for fluorescent staining (Jackson Immunoresearch) were donkey anti-rabbit antibodies conjugated with Alexa488, Cy3 or Alexa647, donkey anti-goat antibodies conjugated with Alexa488, Cy3 or Alexa647, donkey anti-chicken antibodies conjugated with Alexa488, Cy3 or Alexa647 and donkey anti-mouse antibody conjugated with Cy3. Fluorescent slides were covered with Fluoromount-G (SouthernBiotech). DAB slides were covered with DPX (Sigma). Confocal images were taken on NikonA1RGaAsP inverted microscope. Bright field images were captured using an Olympus BX50 microscope.

### Generation of Gsx1 antibody

The Gsx1 antibody was raised in guinea pigs against the C-terminal peptide of human Gsx1, SAPQGCKCASLSSAKCSEDDDELPMSPSSSGKDDRDLTVTP (service provided by Pierce Custom Services, a subdivision of Life Technologies/Thermo-Fisher Pierce) and used at 1:4000 dilution. Heated citrate retrieval solution was used to enhance the staining of Gsx1. This antibody was generated in Dr. Ronald Waclaw’s lab at Cincinnati Children’s Hospital Medical Center and its specificity was confirmed on *Gsx1* mutants [39] at E18.5 (shown in Additional file 1: Figure S1). Control staining for Gsx1 protein in the ventral most LGE progenitors and in the developing hypothalamus (Additional file 1: Figure S1) is similar to previously characterized *Gsx1* gene expression [28, 30, 32, 41].

### Bromodeoxyuridine (BrdU) labeling

Pregnant females were given one dose of BrdU (Sigma-Aldrich) (100 mg/kg) by intraperitoneal injection with embryos at E15.5 stage. Embryos were collected 1 h or 24 h later to examine proliferation and cell cycle retention [42]. Embryos were processed as described above. Cryosectioned 12 μm tissues were treated 50 min for antigen-retrieval with 2 N HCl at room temperature followed by PBS washes and incubation in rat anti-BrdU (1:200, BioRad) overnight.

### In situ hybridization

In situ hybridization was performed as previously described [43]. Digoxigenin-labeled antisense probe against 3′-UTR of *Zic3* mRNA [38] was used on 12 μm cryosections from E18.5 embryos.

### Quantification

For the quantification of tdTomato fate mapped and *Zic3-lacZ* expressing OB interneuron subtypes in the GL, three animals were analyzed on the medial side of the OB. Three control and three *Gsx2* germline mutant (KO) embryos were analyzed for *Gsx2* KO phenotype. Quantification of the embryonic (E18.5) OB phenotype in *Gsx2* conditional mutants (cKOs) was performed on three controls and three *Gsx2* cKOs. Quantification of the P14 OB phenotype in *Gsx2* cKOs was performed using Imaris (Bitplane) to analyze the GL on the medial side of OB of controls (*n* = 5) and *Gsx2* cKOs (*n* = 5). Quantitative results were presented as mean ± standard error of the mean (s.e.m.). Statistical significance was determined using the Student’s *t*-test.

## Results

### *Zic3-lacZ* marks a subpopulation of septal cells and their OB derivatives

**Fig. 1:**
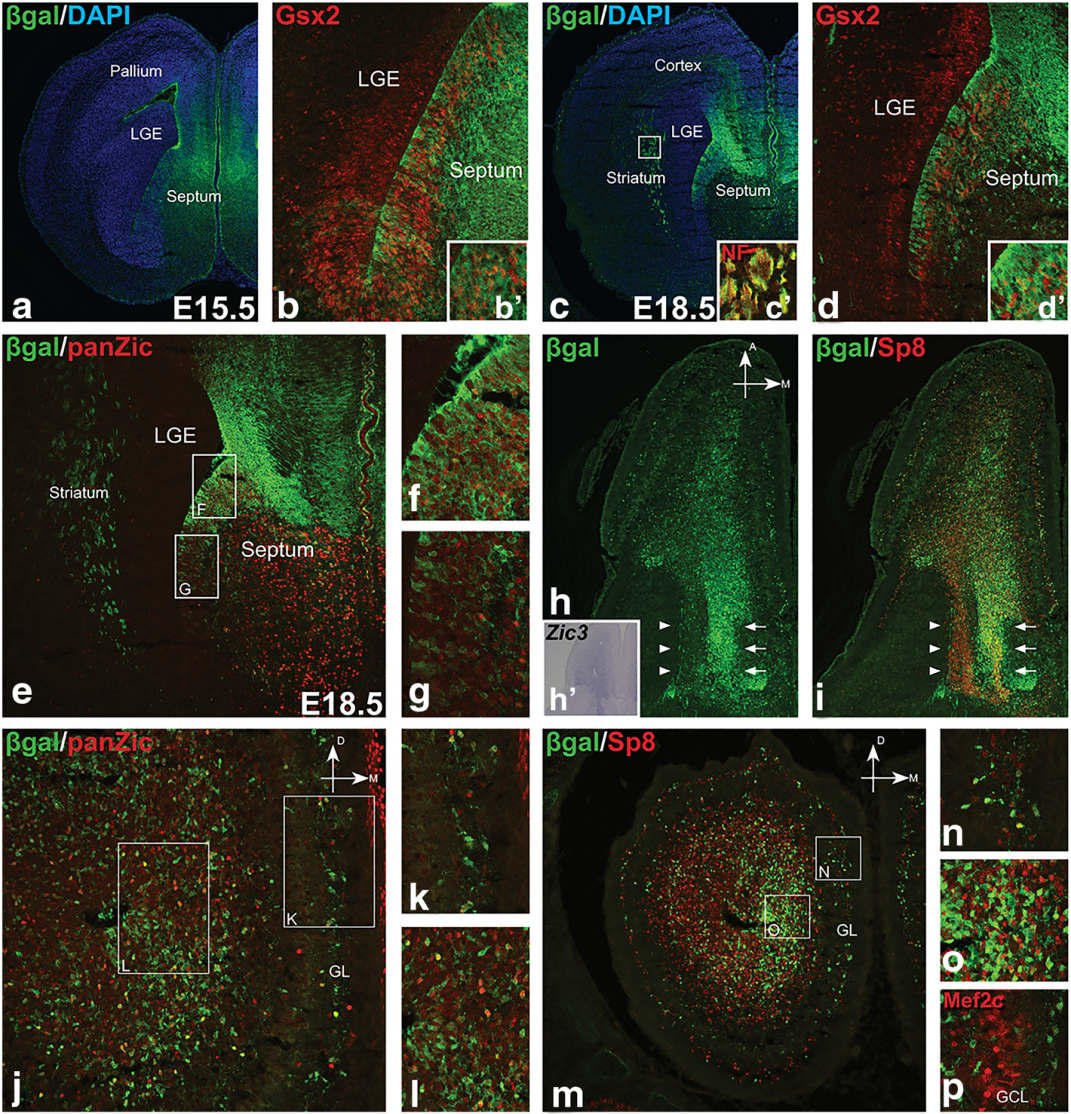
βgal from *Zic3-lacZ* marks the septal primordia and OB cells originating from septum. (**a**-**d**) βgal was enriched in the E15.5 and E18.5 septum and co-localized with Gsx2 within VZ progenitors (**b**, **d**). Insets in (**b**) and (**d**) show high magnification views of septum (*b*’) and (*d*’). Inset in (**c**) shows βgal staining in the E18.5 striatum was found in axons ascending from diencephalon as evidenced by co-labeling with neurofilament (*c*’). (**e**-**g**) βgal from *Zic3-lacZ* displayed a dorsal to ventral gradient, similar to Zic proteins stained by panZic antibody, in the E18.5 septum. Boxes in (**e**) represent high magnification views in (**f**) and (**g**). (**h**, **i**) Horizontal section of E18.5 brain showed βgal^+^ cells migrating to the OB along the RMS and expressing Sp8 on the medial side (indicated by *arrows*) in a pattern similar to endogenous *Zic3* gene expression shown in the inset (*h*’), whereas very few βgal^+^ cells were on the lateral side despite the presence of migrating Sp8^+^ cells (indicated by *arrowheads*). (**J**-**L**) Many βgal^+^ and Zic^+^ cells were found in the E18.5 OB in regions including the forming GL (**k**) and the germinal zone (**l**). Boxes in (**j**) represent high magnification views in (**k**) and (**l**). Most βgal^+^ cells were Zic^+^, while many Zic^+^ cells were βgal^−^. (**m**-**o**) Many βgal^+^ cells in the forming GL (**n**) and germinal zone (**o**) of the OB co-expressed Sp8, constituting a subpopulation of Sp8^+^ interneurons. Boxes in (**m**) represent high magnification views in (**n**) and (**o**). (**p**) Mef2c^+^ granule cells in the E18.5 OB did not express βgal

*Zic (1-4)* genes are highly expressed in medial telencephalic progenitors, including those in the septum, but not in lateral progenitors, and have been implicated in OB interneuron development [44, 45]. Additionally, recent tamoxifen-regulated fate mapping studies using *Fgf8* ^*CreER/+*^ and *Fgf17* ^*CreER/+*^ alleles have revealed that, at early telencephalic stages, these medial telencephalic regions give rise to the septum, as well as a subpopulation of OB interneurons [46]. To label septal progenitors and their progeny, we utilized a *lacZ* reporter under the control of *cis*-regulatory modules of the *Zic3* gene. The *Zic3-lacZ* transgenic line was generated by inserting *lacZ* into the *Zic3* locus in a bacterial artificial chromosome (BAC) [38]. We observed that β-galactosidase (βgal) from *Zic3-lacZ* transgene was largely restricted to the Zic^+^ medial telencephalon, with only a few scattered cells occasionally observed in the ventricular zone (VZ) of the rostroventral LGE (Fig. 1A-G). We also detected βgal staining in the striatum from E18.5 onward; however, this staining marked axons likely from the diencephalon, as confirmed by Neurofilament-M double staining (Fig. 1C, inset and data not shown). In the medial progenitors, a gradient of βgal staining was noticeable in the septal VZ, which was more uniform in the dorsal septum (Fig. 1A-G). Overall, the *lacZ* expression pattern was consistent with that of the endogenous *Zic3* gene and overall Zic proteins (Fig. 1E-G) [44]. At E15.5, βgal was robustly expressed in the septum. Indeed, we found many Gsx2^+^ progenitor cells in the septal VZ coexpressing βgal (Fig. 1A, B). Similarly, many Gsx2^+^ progenitor cells were βgal^+^ in the E18.5 septum (Fig. 1C, D). These data suggested that *Zic3-lacZ* could be an effective tool to mark Gsx2^+^ progenitor cells in the septum and possibly their progeny. The septum is an important source of many neuronal cell types, including OB interneurons [9, 10, 14, 16, 33, 44, 46]. Moreover, *Zic3* expressing cells originating from the septum have been shown to migrate toward the OB [44]. To understand whether medial progenitors in the septum contribute to the developing OB, we immunostained horizontal sections from E18.5 *Zic3-lacZ* septum and OB with antibodies against βgal and Sp8 and found many βgal^+^ cells expressing Sp8 migrating anteriorly from the septum to the OB in a pattern similar to *Zic3* gene expression (inset in Fig. 1H). We also found many βgal^+^ cells in the germinal zone of the E18.5 OB, with a strong bias towards its medial side; moreover, in the GL we found βgal^+^ cells distributed around the OB with a concentration on the medial side (Fig. 1J-O). Many βgal^+^ cells in the E18.5 OB were also labeled by panZic and expressed the transcription factor Sp8, suggesting they were indeed OB interneurons (Fig. 1J-O). Interestingly, few, if any, βgal^+^ cells were observed to express the GCL OB interneuron marker Mef2c [47, 48] (Fig. 1P). Thus, *Zic3-lacZ* mice provide a useful tool to study the OB interneuron progeny of septal progenitors.

### Differential contributions to OB interneuron subtypes from septal and LGE progenitors

**Fig. 2:**
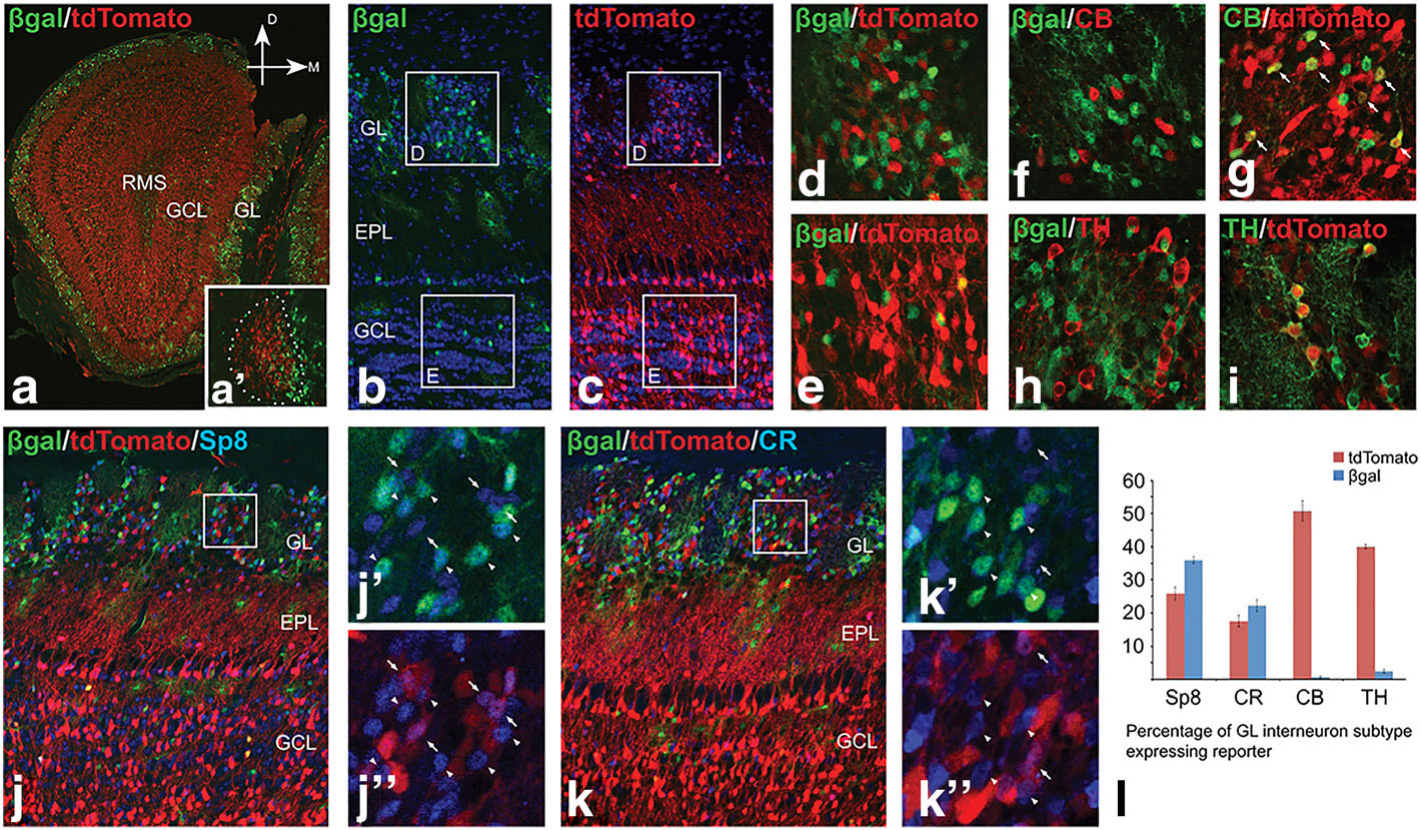
Septum and LGE contribute to OB interneuron subtypes differently. (**a**) βgal^+^ (septum-derived) and tdTomato^+^ (LGE-derived) cells distributed around the P14 OB, despite their bias towards medial and lateral migration, respectively (inset of RMS (*a*’)). (**b**-**e**) βgal and tdTomato expressing cells were largely separated and showed distinct layer localization in the P14 OB. Boxes in (**b**) and (**c**) represent high magnification views in (**d**) and (**e**). βgal^+^ cells were enriched in the GL (**b**, **d**) whereas tdTomato^+^ cells were abundant in both GL and GCL (**c**-**e**). (**f**, **g**) LGE (tdTomato^+^) but not septum (βgal^+^) progenitors gave rise to CB^+^ PGCs. (**h**, **i**) TH^+^ PGCs did not originate from septum. Instead, many of them were generated from the LGE. (**j**, **k**) Both LGE (tdTomato^+^ cells indicated by *arrows*) and septum (βgal^+^ cells indicated by *arrowheads*) contributed to Sp8^+^ and CR^+^ PGCs. Boxes in (**j**) and (**k**) represent high magnification views in (*j*’, *j*”, *k*’, *k*”) respectively. (**l**) Quantification of each PGC subtypes from LGE- versus septum-lineages. Data represent the mean ± s.e.m.

OB interneurons are physiologically and biochemically diverse and can be categorized into different subtypes based on various criteria including the biochemical markers they express, OB layer they occupy as well as neuronal connections they make [3, 4, 5, 6, 7, 19]. Previous studies have suggested that the biochemical and morphological diversity of OB interneurons is determined by the location of the neural stem cell domains from which they originate [9, 10, 16, 49]. To further explore the OB interneuron subtypes derived from embryonic and neonatal neural progenitor cells of distinct domains, we utilized *Zic3-lacZ* transgene as a genetic short-term fate map tool to study OB interneuron subtypes from the septum. In addition, we recently generated the *Gsx2e-CIE* transgenic line, which robustly fate-maps LGE (but not septal) derivatives [36] and therefore allows us to assess LGE-derived OB interneuron subtypes in the same tissue as the *Zic3-lacZ* labeled interneurons. Indeed, we crossed the *Gsx2e-CIE* mice with a *Rosa* ^*tdTomato*^ (*Ai14*) reporter line [37] in the presence of *Zic3-lacZ* and found that LGE-lineage cells fate-mapped by tdTomato (hereafter referred to as tdTomato^+^ cells) and βgal from *Zic3-lacZ* were largely non-overlapping within the OB at P14, when the peak of neonatal OB interneuron neurogenesis occurs [7, 15] (Fig. 2A-E). Interestingly, although the βgal^+^ and tdTomato^+^ cells were mixed within the GL of the OB, βgal^+^ and tdTomato^+^ neuroblasts in the RMS remained on the side from which they originated (i.e. medial and lateral, respectively) (Fig. 2A, inset). In the P14 OB, we found βgal^+^ cells were largely confined to the GL, with only scattered βgal^+^ cells in other OB layers, including the external plexiform layer (EPL) and the GCL (Fig. 2B). In fact, about 29.3 ± 1.3% of the GL cells were βgal^+^, whereas only 4.7 ± 1.0% of the cells in GCL expressed βgal. This is in line with the observation that few, if any, of the Mef2c^+^ granule cells were βgal^+^ in the E18.5 OB (Fig. 1P). In contrast, numerous LGE-derived tdTomato^+^ cells were found in both the GL and GCL (Fig. 2C-E) [36]. The GL enrichment of βgal^+^ cells suggested that the GL was a major destination of septum-derived OB cells and therefore represented the focus of the present study. To examine the neuronal subtype identity of the βgal^+^ cells in the GL, we immunostained the P14 OB with antibodies against markers of periglomerular interneuron subtypes, CB, CR and TH and found virtually no CB^+^ neurons were labeled by βgal (Fig. 2F, L). In contrast, about 50.1 ± 3.0% of the CB^+^ cells were tdTomato^+^ (Fig. 2G, L), suggesting that a significant portion of CB^+^ interneurons were derived from the LGE but not the septum. Similarly, we found few, if any, TH^+^ (i.e. dopaminergic) neurons were βgal^+^, whereas 40 ± 0.6% of them were fate-mapped by tdTomato (Fig. 2H, I, L), indicating that TH^+^ OB interneurons are not septum-derived and that the LGE serves as an important source for this neuronal subtype. The zinc finger transcription factor, Sp8, is expressed by many interneurons residing in both the GL and GCL [20]. We found that 85.7 ± 2.6% of the βgal^+^ cells in the GL were Sp8^+^ (Fig. 2J). A small portion of βgal^+^ cells were Sp8^−^ and displayed glial morphology together with immunoreactivity for the astrocyte marker GFAP (data not shown). The βgal^+^Sp8^+^ double positive cells comprised about one third (36.0%) of the total Sp8^+^ cells in the GL, whereas about 25.9 ± 2.0% of the Sp8^+^ GL neurons were tdTomato^+^ (i.e. LGE-derivatives) (Fig. 2J, L). Many of the Sp8^+^ cells in the GL express the calcium binding protein CR [19, 20], and CR^+^ interneurons have been suggested to arise predominantly from the septum at postnatal time points [9, 10]. Indeed, we found 22.2 ± 1.8% of the CR^+^ cells in the GL were βgal^+^, accounting for about 30.5 ± 4.0% of the βgal^+^ cells, supporting a septal origin for at least a portion of this OB interneuron subtype (Fig. 2K, L). However, we also observed that about 17.6 ± 1.7% CR^+^ interneurons were tdTomato^+^, indicating that at least a subpopulation of these interneurons originate from the LGE (Fig. 2K, L). Taken together, these data support the notion that the LGE, more specifically the dLGE, gives rise to all three subtypes (i.e. TH, CB and CR) of GL interneurons, while the septum largely contributes to the CR subtype. Moreover, the dLGE provides neurons to populate both the GL and GCL, while the septum-derived progeny are specifically targeted to the GL.

### Impaired septum-derived OB neurogenesis in germline *Gsx2* knockouts

**Fig. 3:**
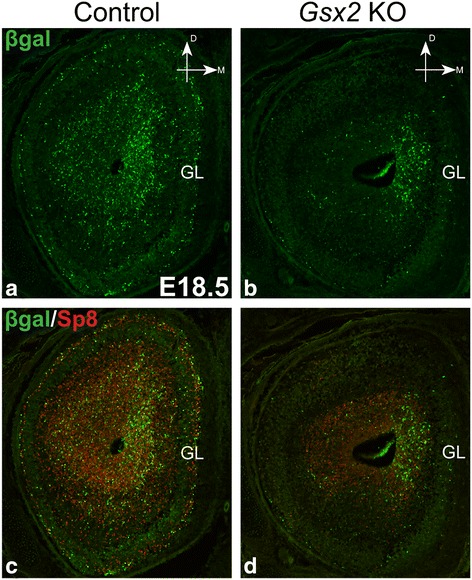
*Gsx2* germline knockout (KO) impairs the generation of septum-derived OB cells. **a**, **b** *Gsx2* KO carrying the *Zic3-lacZ* allele showed a dramatic loss of βgal^+^ cells in the E18.5 OB. **c**, **d** Septum-derived (i.e. βgal^+^) and non-septum-derived (i.e. βgal^−^) Sp8^+^ interneurons were also severely compromised in the E18.5 *Gsx2* KO OB

The homeobox gene *Gsx2* is expressed at various levels by many VZ progenitor cells in the embryonic ventral telencephalon, including the LGE, MGE and septum [29, 40, 50], as well as in the postnatal dorsolateral SVZ (dlSVZ), a derivative of the embryonic dLGE [48]. Previous studies revealed that Gsx2 is critical for the normal generation of many LGE-derived cell types including OB interneurons, amygdalar intercalated cells and striatal projection neurons [20, 27, 28, 29, 30, 31, 32, 40, 51]. Despite that altered transcriptional profiles have been reported in the *Gsx2*-deficient septum [34], the role of Gsx2 in specifying septum-derived OB interneurons has not been well characterized. To test the requirement of *Gsx2* in the generation of OB cells from septum, we crossed the *Zic3-lacZ* allele onto *Gsx2* germline knockout (*Gsx2* KO, *Gsx2* ^*RA/EGFP*^ or *Gsx2* ^*RA/RA*^) and control (*Gsx2* ^*RA/+*^ or *Gsx2* ^*EGFP/+*^) mice [31, 32]. We found reduced numbers of βgal^+^ cells in both the germinal zone and GL of the *Gsx2* KO bulb at E18.5 (Fig. 3a, b). In agreement with previous studies [31, 32], we also observed a 70% reduction (control: 96.9 ± 11.4 versus *Gsx2* KO: 26.8 ± 7.0 cells, *p* = 0.006) of Sp8^+^ interneurons in the forming GL of the *Gsx2* KO OB, which was concomitant with about a 60% loss (control: 21.3 ± 3.4 versus *Gsx2* KO: 7.8 ± 3.3 cells, *p* = 0.045) of the βgal^+^ (i.e. septum-derived) population (Fig. 3c, d). These data supported the notion that *Gsx2* is critical for the normal generation of septum-derived OB interneurons as is the case for the LGE-derived populations [31, 32].

### Reduced septum-derived interneurons in the *Olig2*^*Cre/+*^ driven *Gsx2* cKO OB

**Fig. 4:**
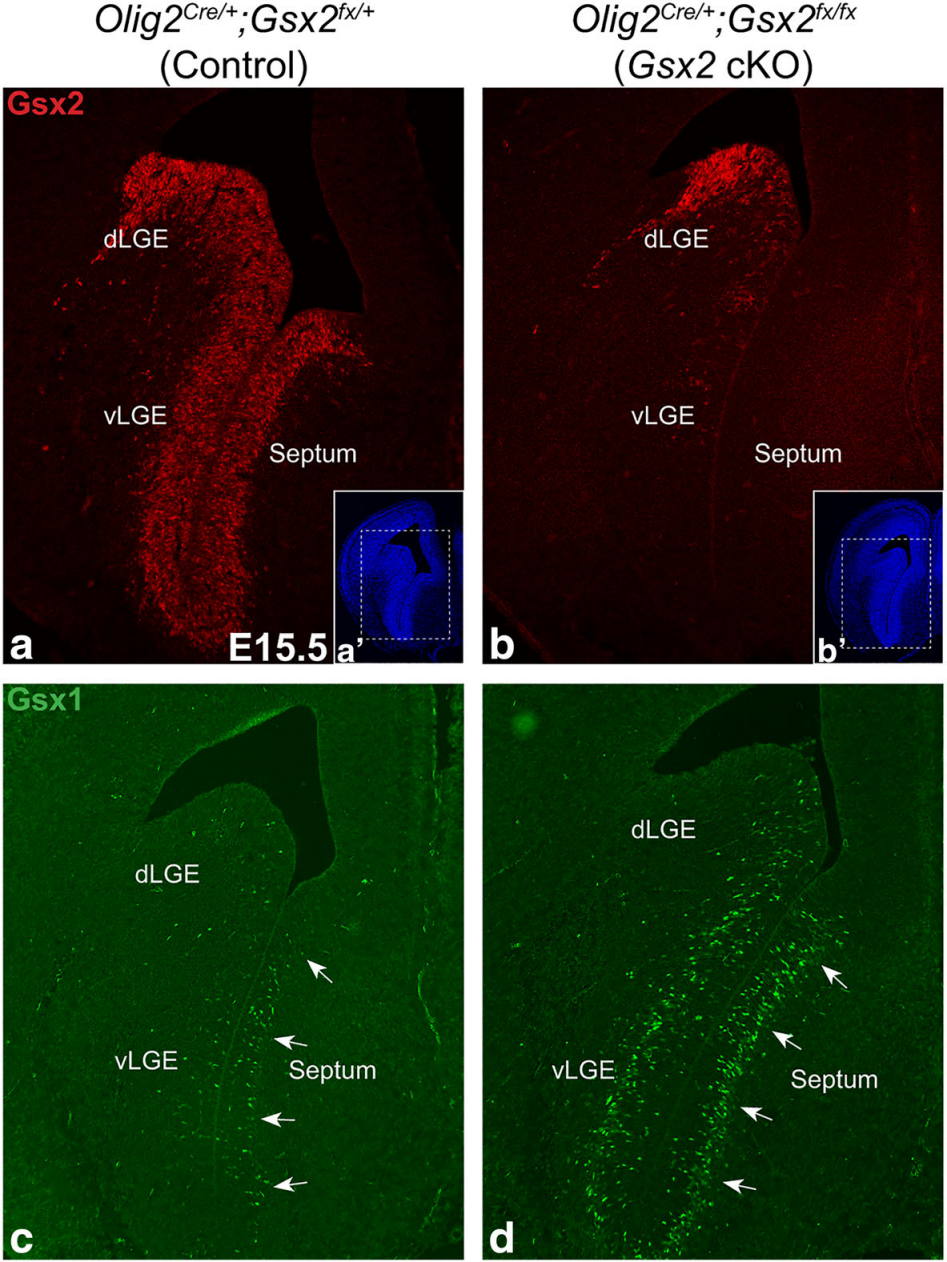
Conditional inactivation of *Gsx2* in the septum by *Olig2* ^*Cre/+*^. (**a**, **b**) Gsx2 protein was lost in the septum and vLGE of the E15.5 *Gsx2* cKO embryos, however its expression was relatively normal in the *Gsx2* cKO dLGE. Insets in (**a**) and (**b**) represent low magnification DAPI stains (*a*’, *b*’). Dashed boxes in (*a*’) and (*b*’) represent the magnification in (**a**) and (**b**). (**c**, **d**) Accordingly, the Gsx2 family member, Gsx1 was found upregulated in the E15.5 *Gsx2* cKO septum and vLGE

**Fig. 5:**
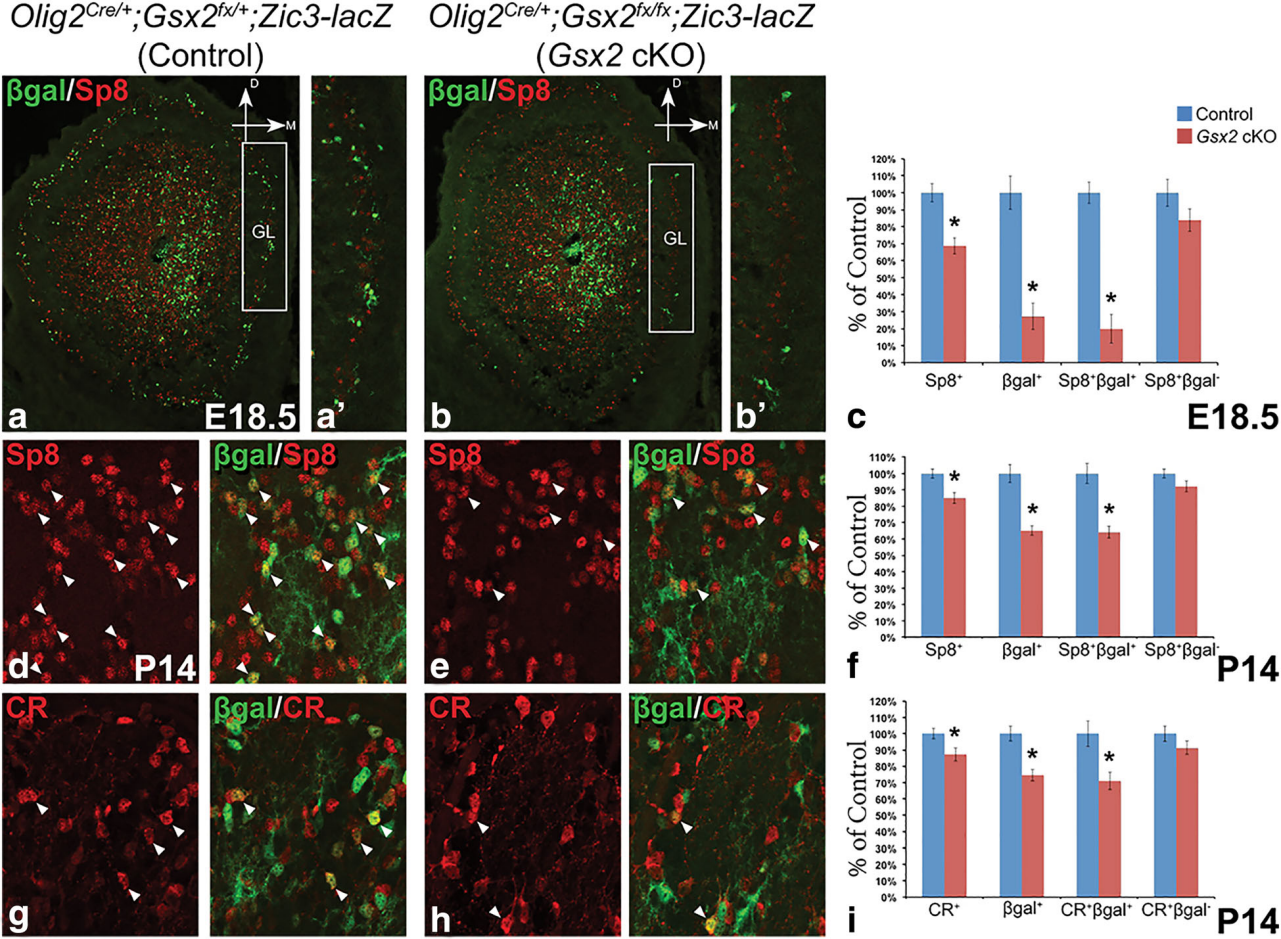
Impairment of septum-derived PGCs in the *Gsx2* cKO OB. (**a**-**c**) Septum-derived GL cells marked by βgal were reduced, leading to a significant loss of septum-originated Sp8^+^ interneurons (i.e. βgal^+^) and a milder reduction of total Sp8^+^ PGCs in the E18.5 *Gsx2* cKO OB, whereas LGE-derived Sp8^+^ interneurons (i.e. βgal^−^) were largely normal. Boxes in (**a**) and (**b**) indicate the GL area shown in (*a*’) and (*b*’) respectively. (**d**-**f**) Sp8^+^ PGCs were reduced in the P14 *Gsx2* cKO OB, primarily due to the reduced number of the septum-derived (i.e. βgal^+^) interneurons. *Arrowheads* indicate Sp8^+^βgal^+^ cells that originated from the septum. (**g**-**i**) P14 *Gsx2* cKO OB showed reduced number of CR^+^ PGCs, particularly those βgal-expressing ones generated from the septum. Other CR^+^ PGCs (i.e. βgal^−^), presumably originating from other regions including LGE, were largely intact. Data represent the mean ± s.e.m. *p < 0.05

As shown above, reduction in the numbers of βgal^+^ and Sp8^+^ cells was observed in the E18.5 *Gsx2* mutant OB. However, germline *Gsx2* mutant mice die at birth [52], preventing further analysis of the impact on the generation of mature OB phenotypes. *Olig2* is robustly expressed not only by cells of the oligodendrocyte lineage [53], but also by many neurogenic progenitor cells in the VZ of ventral telencephalic regions including the MGE, LGE and the septum [54, 55, 56]. Within the LGE, *Olig2* is highly expressed in the VZ cells of the ventral (v)LGE while its expression in the dLGE is quite limited. Therefore we took advantage of an *Olig2* ^*Cre/+*^ line [35] to selectively knockout *Gsx2* in the septum and vLGE, while leaving it largely intact within the dLGE, as previously published [57]. We found a complete loss of Gsx2 in the E15.5 *Olig2* ^*Cre/+*^ *;Gsx2* ^*fx/fx*^ (*Gsx2* cKO) septum and vLGE (Fig. 4B) in comparison to *Olig2* ^*Cre/+*^ *;Gsx2* ^*fx/+*^ (control) which show robust Gsx2 expression in septum and throughout the LGE (Fig. 4A). The expression of Gsx2 in the dLGE of the *Gsx2* cKOs was largely intact (Fig. 4B). We observed a robust upregulation of the closely related family member, Gsx1, in the *Gsx2* cKO septum and vLGE (Fig. 4C, D). This is in line with previous studies that show Gsx1 can partially compensate for the loss of Gsx2 in the LGE [28, 30]. To investigate whether medial derived OB interneurons are compromised when *Gsx2* is inactivated in the septum, we used *Zic3-lacZ* as a reporter and immunostained E18.5 control and *Gsx2* cKO OB with antibodies against Sp8 and βgal. In agreement with the observation in E18.5 *Gsx2* KO OB, we found the number of βgal^+^ cells was reduced by 73% in the *Gsx2* cKO OB, as compared to controls (control: 26.1 ± 2.6 versus cKO: 7.1 ± 2.0 cells per field, *p* = 0.001) (compare Fig. 5B with A). The total number of Sp8^+^ cells in the *Gsx2* cKO GL was reduced by 31% (control: 70.4 ± 3.8 versus cKO: 48.3 ± 3.3 cells per field, *p* = 0.023) (Fig. 5A-C). Importantly, the number of Sp8^+^βgal^+^ double labeled cells (i.e. septum-derived) in the *Gsx2* cKO GL was reduced by approximately 80% from control (control: 16.7 ± 1.1 versus cKO: 3.3 ± 1.4 cells per field, *p* = 0.003) (Fig. 5A-C). In contrast, the number of Sp8^+^βgal^−^ GL cells in the *Gsx2* cKO OB, which are presumably derived largely from the dLGE, was not significantly different from that in the control (control: 53.7 ± 4.3 versus cKO: 45.0 ± 3.5 cells per field, *p* = 0.11) (Fig. 5A-C).

At E18.5 the OB is undergoing development, and many interneurons that are specified during embryonic stages have not yet reached their final destination in the bulb and begun to express mature biochemical markers (e.g. CB, CR and TH). In addition, only a portion (about 20%) of the OB interneurons in the rodent are born embryonically [15, 58], a significant amount of them are generated during the early postnatal stage [7, 15]. Unlike *Gsx2* germline knockouts, *Olig2* ^*Cre/+*^ *;Gsx2* ^*fx/fx*^ (*Gsx2* cKO) mice are viable after birth, allowing us to assess interneuron phenotypes in the postnatal OB. Therefore we generated P14 *Olig2* ^*Cre/+*^ *;Gsx2* ^*fx/+*^ *;Zic3-lacZ* (control) and *Olig2* ^*Cre/+*^ *;Gsx2* ^*fx/fx*^ *;Zic3-lacZ* (*Gsx2* cKO) mice and immunostained their OB with antibodies against different OB interneuron markers. We found a 15% reduction (control: 1987.8 ± 54.4 versus cKO: 1691.8 ± 67.5 cells/mm^2^, *p* = 0.009) of Sp8^+^ interneurons along with a significant reduction (35%) of βgal^+^ cells (control: 595.3 ± 32.1 versus cKO: 387.7 ± 17.3 cells/mm^2^, *p* = 0.0005) in the GL of the *Gsx2* cKO OB (Fig. 5D-F). Furthermore, the compromised Sp8^+^ population was primarily due to the loss of septum-derived Sp8^+^ periglomerular cells, as a 36% reduction (control: 506.1 ± 30.5 versus cKO: 324.5 ± 18.4 cells/mm^2^, *p* = 0.001) of Sp8^+^βgal^+^ double labeled cells in the GL was observed (Fig. 5D-F). In contrast, the Sp8^+^βgal^−^ GL cells (presumably dLGE-derived) in the mutants were not significantly different from control (control: 1481.7 ± 40.0 versus cKO: 1367.3 ± 50.2 cells/mm^2^, *p* = 0.11) (Fig. 5D-F). The postnatal septum is known to give rise to CR^+^ interneurons in the GL (Fig. 2K, L) [9, 10, 16]. We also detected a 13% reduction (control: 849.9 ± 26.7 versus cKO: 741.5 ± 34.2 cells/mm^2^, *p* = 0.037) of CR^+^ interneurons in the GL of the *Gsx2* cKO OB (Fig. 5G-I). Importantly, a 29% reduction of the septum-derived CR^+^βgal^+^ cells was observed in the GL of the *Gsx2* cKOs (control: 171.2 ± 13.5 versus cKO: 121.7 ± 9.2 cells/mm^2^, *p* = 0.016), whereas the CR^+^βgal^−^ population (i.e. dLGE-derived) was not significantly different from that in control (control: 678.7 ± 31.9 versus cKO: 619.8 ± 27.7 cells/mm^2^, *p* = 0.2) (Fig. 5G-I). Few, if any, of the CB^+^ or TH^+^ interneurons in the GL originate from the septum (see Fig. 2F, H, L). Accordingly, normal numbers of CB^+^ and TH^+^ GL interneurons were observed in the *Gsx2* cKO OB (data not shown).

### Conditional knockout of *Gsx2* in the septum leads to development defects

**Fig. 6:**
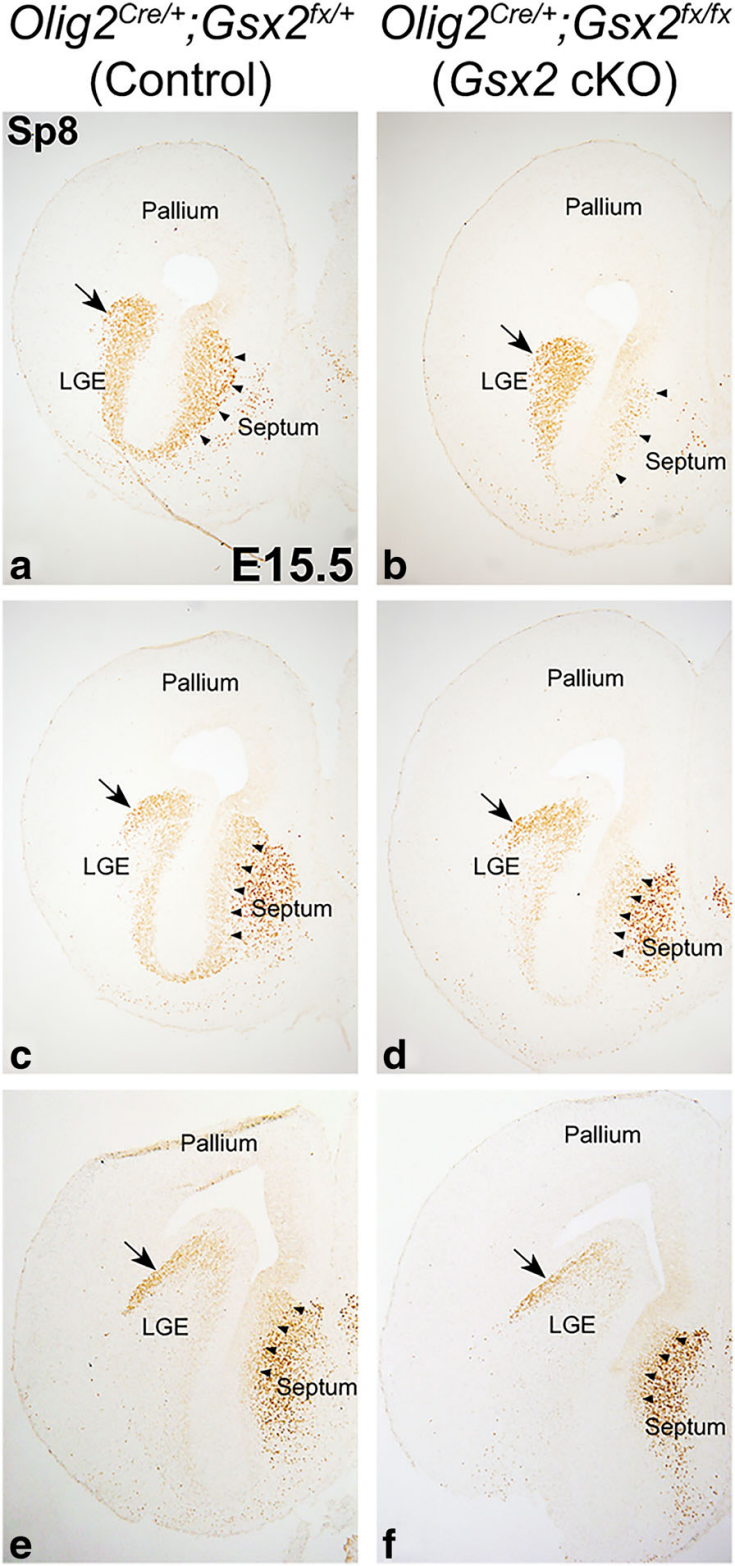
Reduced neuroblasts in the *Gsx2* cKO septum. The number of Sp8^+^ neuroblasts in the E15.5 *Gsx2* cKO septum was impaired, mostly at a rostral levels (compare (**a**, **c**) to (**b**, **d**) indicated by *arrowheads*), whereas Sp8 staining in the caudal septum was relatively normal (compare (**e**) and (**f**), indicated by *arrowheads*). In contrast, Sp8^+^ neuroblasts generated in the LGE were largely unchanged (indicated by *arrows* from (**a**-**f**))

**Fig. 7:**
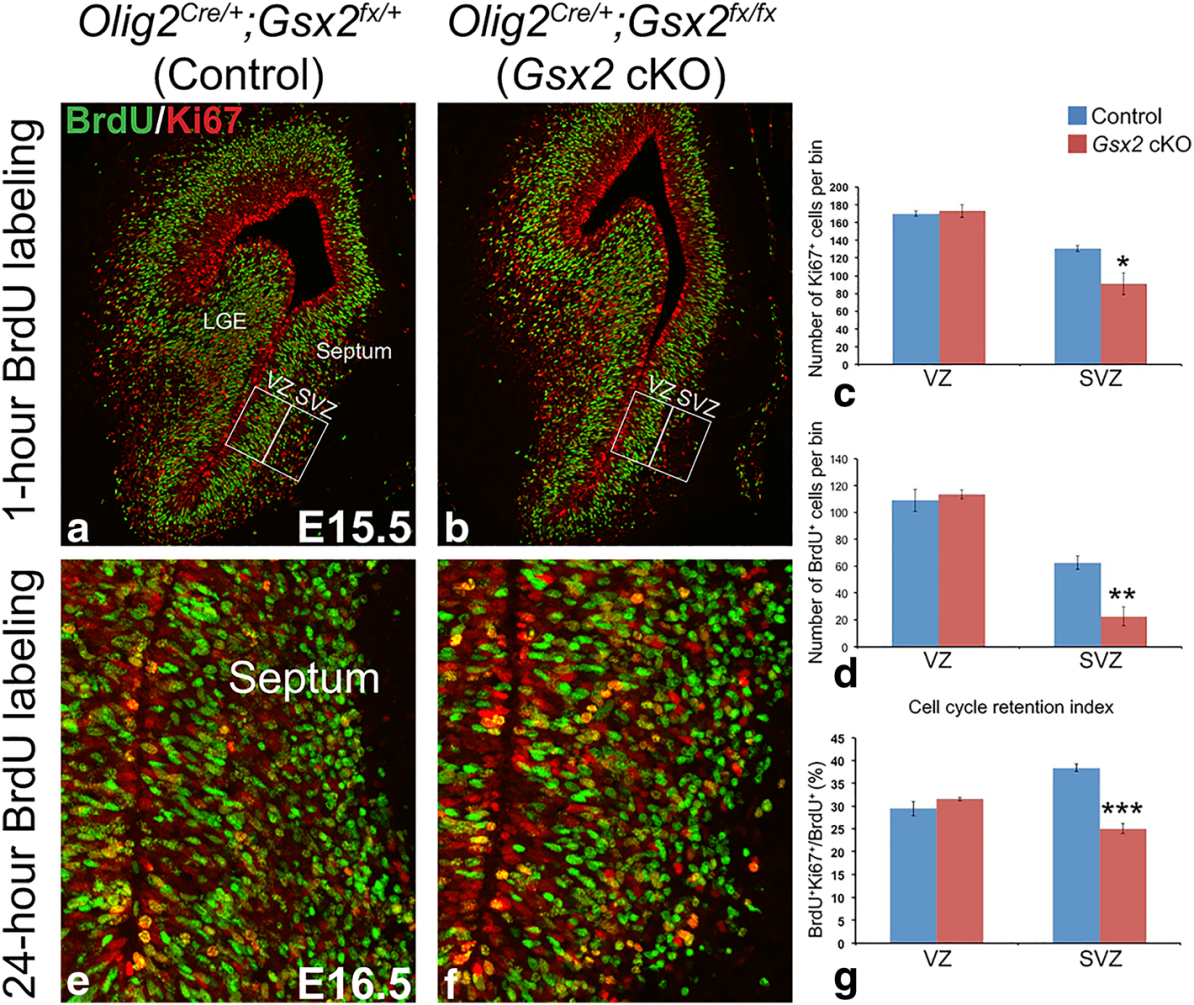
Impaired cell proliferation in the *Gsx2* cKO septum. **a**-**d** *Gsx2* cKO septum showed reduced cell proliferation in the SVZ, but not in the VZ, at E15.5, as revealed by Ki67 and BrdU staining after one-hour BrdU labeling. The VZ was defined according to the apical ventricular surface and the basal region where S-phase BrdU^+^ nuclei were enriched. The same size box was placed adjacent to the VZ to represent the SVZ. **e**-**g** Cell cycle retention index was relatively normal in the VZ but decreased in the SVZ of the *Gsx2* cKO septum 24 h after E15.5 BrdU administration. Data represent the mean ± s.e.m. *p < 0.05, **p < 0.01, ***p < 0.005

Gsx2 has been implicated in the generation of OB interneurons from the dLGE by regulating the temporal specification of Sp8^+^ neuroblasts [20, 31, 32, 50, 57]. To determine whether *Gsx2* inactivation by *Olig2* ^*Cre/+*^ impairs the normal specification and/or generation of Sp8^+^ neuroblasts in the developing septum, we analyzed E15.5 control and *Gsx2* cKO embryos. Similar to what was found in the *Gsx2*-deficient LGE [28], we observed a dramatic loss of Sp8^+^ cells in the *Gsx2* cKO septum, particularly at the rostral level where a large amount of Sp8^+^ cells were present in the control septum (arrowheads in Fig. 6). In contrast, Sp8 staining in the *Gsx2* cKO dLGE was comparable to that in the control embryos (arrows in Fig. 6), consistent with the fact that Gsx2 expression remains in the dLGE of conditional mutants. Gsx2 is critical for maintaining LGE progenitor cells in an undifferentiated state and promoting their self-renewal capacity [50]. In the absence of Gsx2, the embryonic LGE displays compromised cell proliferation and, thus, fails to establish a normal SVZ [28]. To investigate whether Gsx2 regulates proper cell proliferation in the developing septum, we pulsed pregnant females with one dose of BrdU at E15.5 and collected embryos 1 h after BrdU administration to label cells in S-phase. Double staining for BrdU and the cell proliferation marker Ki67 demonstrated that cell proliferation in the septal VZ of *Gsx2* cKO embryos was indistinguishable from that in the control (Fig. 7a-d). However, the numbers of Ki67^+^ and BrdU^+^ cells in the SVZ were dramatically reduced in the *Gsx2* cKO septum (Fig. 7a-d), suggesting the impairment of the formation of the secondary proliferative zone (i.e. SVZ) in the *Gsx2*-deficient septum. To determine whether cell cycle exit is altered in the *Gsx2* cKO septum, one dose of BrdU was given at E15.5 and embryos were collected 24 h later [42]. Again, we performed double immunofluorescent staining for BrdU and Ki67 and calculated cell cycle retention index, measured by the percentage of BrdU^+^ cells labeled at E15.5 remaining in cell cycle (i.e. BrdU^+^Ki67^+^/BrdU^+^), in both control and *Gsx2* cKO septum. While the cell cycle retention index in the VZ of control and *Gsx2* cKO septum were comparable (control: 29.4% versus cKO: 31.6%, *p* = 0.26) at E16.5, we found a reduced percentage of BrdU^+^ cells remaining in the cell cycle (i.e. Ki67^+^) within the SVZ of the *Gsx2* cKO septum (control: 38.4% versus cKO: 25.1%, *p* = 0.0006) (Fig. 7e-g). These findings indicate that Gsx2 is required for the correct establishment of a proliferative SVZ in the septum. Moreover, proliferative progenitors in the *Gsx2*-deficient septal SVZ appear to exit the cell cycle prematurely, thus limiting the number of septum-derived OB interneurons, which is in line with the observed OB phenotype in *Gsx2* germline and cKO mutants.

## Discussion

In this study, we investigated the regional contributions to OB interneuron diversity at embryonic and neonatal stages using both long-term and short-term genetic fate-mapping approaches. Our results indicate that the septum contributes to subpopulations of Sp8^+^ and CR^+^ interneurons in the GL but not to their CB^+^ or TH^+^ counterparts. In contrast, the dLGE contributes to all these subtypes, including CB^+^, CR^+^ and TH^+^, as well as large numbers of interneurons that occupy both the GL and GCL. The homeobox gene *Gsx2* has been suggested to be critical for the normal generation of OB interneurons from the embryonic dLGE [31, 32], however, its role in the generation of septum-derived OB interneurons has not been described. By conditionally inactivating *Gsx2* in the septum while largely preserving its expression in the dLGE, we found reduced numbers of septum-derived Sp8^+^ and CR^+^ OB interneurons in the GL. In addition, our data suggest that the OB interneuron defects (reduction of CR^+^ GL cells) observed in *Gsx2* cKO animals result from the impaired proliferation of OB interneuron progenitors in the septal SVZ.

OB interneurons are generated from both dLGE and septum embryonically [8, 14, 16, 20, 31, 33]. It has been suggested that OB interneurons of different subtypes originate from distinct progenitor/neural stem cell domains in the postnatal telencephalon [9, 10, 16]. Although many studies have attempted to address this notion, few genetic approaches have been taken to characterize the origins of OB interneuron subtypes generated during embryonic and the subsequent neonatal periods, when many OB interneurons are born [7, 15]. Recently, a pan-antibody against Zic proteins was used to identify OB interneurons derived from septum [10]. Although panZic^+^ cells were restricted in the E18.5 septum and most of the βgal^+^ cells from the *Zic3-lacZ* lineage were also panZic^+^ in the OB GL, we detected some panZic^+^ cells in the P14 dlSVZ (data not shown). While panZic staining is enriched in the septum and remains a useful tool to examine septum derived cells in postnatal OB, we took a more specific approach using *Zic3* reporter mice expressing βgal from a *Zic3* BAC construct [38]. We found βgal expression to be largely restricted to the medial side of the telencephalon, including the septum, with only occasional clones observed in the ventral-most portion of the embryonic LGE. In addition, no ectopic βgal signal was found in the P14 dlSVZ (data not shown). It was noticeable that βgal from the *Zic3-lacZ* transgene showed a dorsal-high to ventral-low gradient in the septum, and although this pattern was consistent with that of endogenous Zic proteins, its expression was mosaic. Despite this, a considerable portion of the Sp8^+^ cells in the septal SVZ were βgal^+^ (data not shown). In the OB, βgal signal represents both persistent and down-regulated *Zic* gene expression. Most of the βgal^+^ cells were panZic^+^, but some of the cells in which βgal was persistent were not and thus likely represent a short-term fate map of the *Zic* lineage. Thus, *Zic3-lacZ* appears to be a reliable short-term fate map tool for studying septal cells and their OB derivatives.

By combining the *Zic3-lacZ* allele together with the recently characterized LGE driver *Gsx2e-CIE* [36], we assessed the unique contributions to the OB interneuron populations from septum and LGE separately. Our results, at both E18.5 and P14, indicate that septum primarily contributes to OB interneurons (i.e. βgal^+^) in the GL, whereas dLGE-derived interneurons (i.e. tdTomato^+^) were observed to populate all OB layers, including the GL and GCL. Furthermore, our findings indicate that septum-derived OB progenitors are rather limited in potential to generate Sp8^+^ and CR^+^ cells in the GL, whereas the dLGE progenitors give rise to all three major GL subtypes, namely CB^+^, TH^+^ and interestingly also a subpopulation of CR^+^ periglomerular cells. Merkle et al. [9, 10] have previously suggested that the septum represents an exclusive source for CR^+^ OB interneurons. These studies examined the postnatal contributions of septal progenitors, while our study includes both embryonic and early postnatal time points. We found that the septum and dLGE lineages both contribute to Sp8^+^ OB interneurons, among which about half also express CR [20]. In addition, CR^+^ cells have been detected in the E18.5 dLGE, and similar stage *Ascl1* mutants which show aberrant Gsx2 and Sp8 expression in the dLGE, also exhibit enhanced CR staining in the dLGE and OB [32]. The notion that the dLGE represents a separate source of CR^+^ OB interneurons is also supported by our findings that many CR^+^ (βgal^−^) interneurons were present in the *Gsx2* cKO mice, in which Gsx2 remained largely intact in the dLGE. In fact, *Zic3-lacZ* (i.e. septal) and *Gsx2e-CIE* (i.e. dLGE) lineages together account for only about 40% of the total CR^+^ interneurons in the GL. While this could be due, in part, to incomplete recombination/transgene expression, it is also possible that other telencephalic regions may provide a separate subpopulation of CR^+^ OB interneurons. For example, a previous study using a transplantation approach suggested that the pallium is able to contribute to CR^+^ OB interneurons [2].

The specification of diverse OB interneuron subtypes has been suggested to be the result of combinatory effects of many transcription factors referred as transcription factor codes [19, 59]. Our findings that both dLGE and septum give rise to CR^+^ interneurons in the GL which are distinguished, in part, by the expression of Zic proteins in the septum-derived subpopulation raises the question whether CR^+^ interneurons with distinct telencephalic origins are phenotypically/physiologically different.

In the embryonic ventral telencephalon, *Gsx2* is highly expressed by progenitor cells in the VZ of the septum and LGE with a high dorsal to low ventral gradient in each region. We noticed co-localization of βgal from the *Zic3-lacZ* transgene and Gsx2 in the septal VZ as well as maintained βgal expression in the embryonic and postnatal OB which allowed us to use this reporter mouse as a short-term genetic fate map tool to study the contribution of Gsx2^+^ progenitors in the septum to their OB progeny. We found that septum-derived (i.e. βgal-expressing) Sp8^+^ and CR^+^ OB interneurons were reduced in the GL when *Gsx2* was selectively inactivated in the septum using an *Olig2* ^*Cre/+*^ driver. Similarly, we observed decreased panZic-labeled Sp8^+^ cells in the forming GL of the E18.5 *Gsx2* cKO OB (data not shown). Moreover, this phenotype was not due to a lack of Zic proteins or βgal expression, as both remained in the *Gsx2* cKO septal progenitors (data not shown). Therefore, these findings indicate an important role for Gsx2 in regulating normal OB interneuron generation from the septum, specifically the septum-derived CR^+^ periglomerular cells. Interestingly, the reduction of septum-derived Sp8^+^ interneurons in the P14 *Gsx2* cKO OB was not as pronounced as that in the E18.5 OB. This is probably due to the upregulation of *Gsx1*, a family member of *Gsx2*, which has been shown to partially compensate for the loss of *Gsx2* in the LGE via upregulation at the VZ/SVZ boundary [28, 30, 32, 50, 57]. Indeed, Gsx1 upregulation was already detected in E15.5 embryos also at the VZ/SVZ boundary of the *Gsx2* cKO septum. In addition, *Gsx-*independent neurogenesis may also occur in the septum during later developmental stages. In fact, Gsx1 does not compensate for the loss of Gsx2 in the adult dlSVZ [48], suggesting a *Gsx*-independent neurogenesis program for some OB interneuron subtypes. We observed reduced Sp8^+^ cells and impaired cell cycle kinetics in the SVZ of the *Gsx2*-deficient septum of E15.5 embryos. Similar phenotypes were also reported in the *Gsx2* knockout LGE [20, 28, 31, 32, 57]. Interestingly, we found a reduced cell cycle retention index in SVZ but not in VZ progenitors of the *Gsx2* cKO septum. Given that Gsx2 is present in primary progenitor cells (i.e. VZ), our results suggest that Gsx2 regulates OB interneuron generation from the septum by enhancing indirect neurogenesis and/or indirectly influencing the proliferation capacity of basal (i.e. SVZ) progenitors derived from Gsx2 expressing VZ cells. One possible mechanism is that Gsx2 may repress or reduce *Gsx1* expression. Despite that Gsx1 shares similar function with Gsx2 in telencephalic patterning, it promotes progenitor maturation and neurogenesis while Gsx2 helps to maintain progenitors in an undifferentiated state [50]. Therefore, the *Gsx2*-deficient primary progenitors with upregulated Gsx1 may bias towards direct neurogenic cell division and give rise to SVZ cells that exhibit reduced proliferative capacity.

## Conclusions

This study provides evidence that the embryonic/neonatal septum and dLGE contribute to OB interneuron diversity differently. Specifically, the septum contributes CR^+^ cells to the GL, while the dLGE gives rise to interneurons that occupy both the GCL and GL as well as the 3 main subtypes of GL interneurons (CB^+^, CR^+^ and TH^+^). Moreover, our findings indicate a role for Gsx2 in septal VZ progenitors for the generation of proliferative SVZ progenitors specified to generate CR^+^ GL interneurons in the OB.

## Additional file

Additional file 1: Figure S1. Characterization of Gsx1 specific antibody. Immunostaining for Gsx1 in the telencephalon and diencephalon reveals positive cells in the ventral most LGE and developing hypothalamus (*Gsx1* ^*+/+*^) at E18.5 (**A**, **C**). No Gsx1 positive cells are detected in *Gsx1* mutant (*Gsx1* ^*−/−*^) forebrain regions (**B**, **D**). (TIFF 9369 kb)

## Abbreviations

BAC: Bacterial artificial chromosome
BrdU: Bromodeoxyuridine
CB: Calbindin
cKO: Conditional knockout
CR: Calretinin
dLGE: Dorsal lateral ganglionic eminence
E: Embryonic day
GCL: Granule cell layer
GL: Glomerular layer
LGE: Lateral ganglionic eminence
MGE: Medial ganglionic eminence
OB: Olfactory bulb
PGC: Periglomerular cell
RMS: Rostral migratory stream
SVZ: Subventricular zone
TH: Tyrosine hydroxylase
VZ: Ventricular zone
βgal: β-galactosidase
