# Supplementary figures and images for: Septal contributions to olfactory bulb interneuron diversity in the embryonic mouse telencephalon: role of the homeobox gene Gsx2
Source: Neural Dev. 2017 Aug 16;12:13. doi: 10.1186/s13064-017-0090-5 (PMC5559835; doi:10.1186/s13064-017-0090-5)

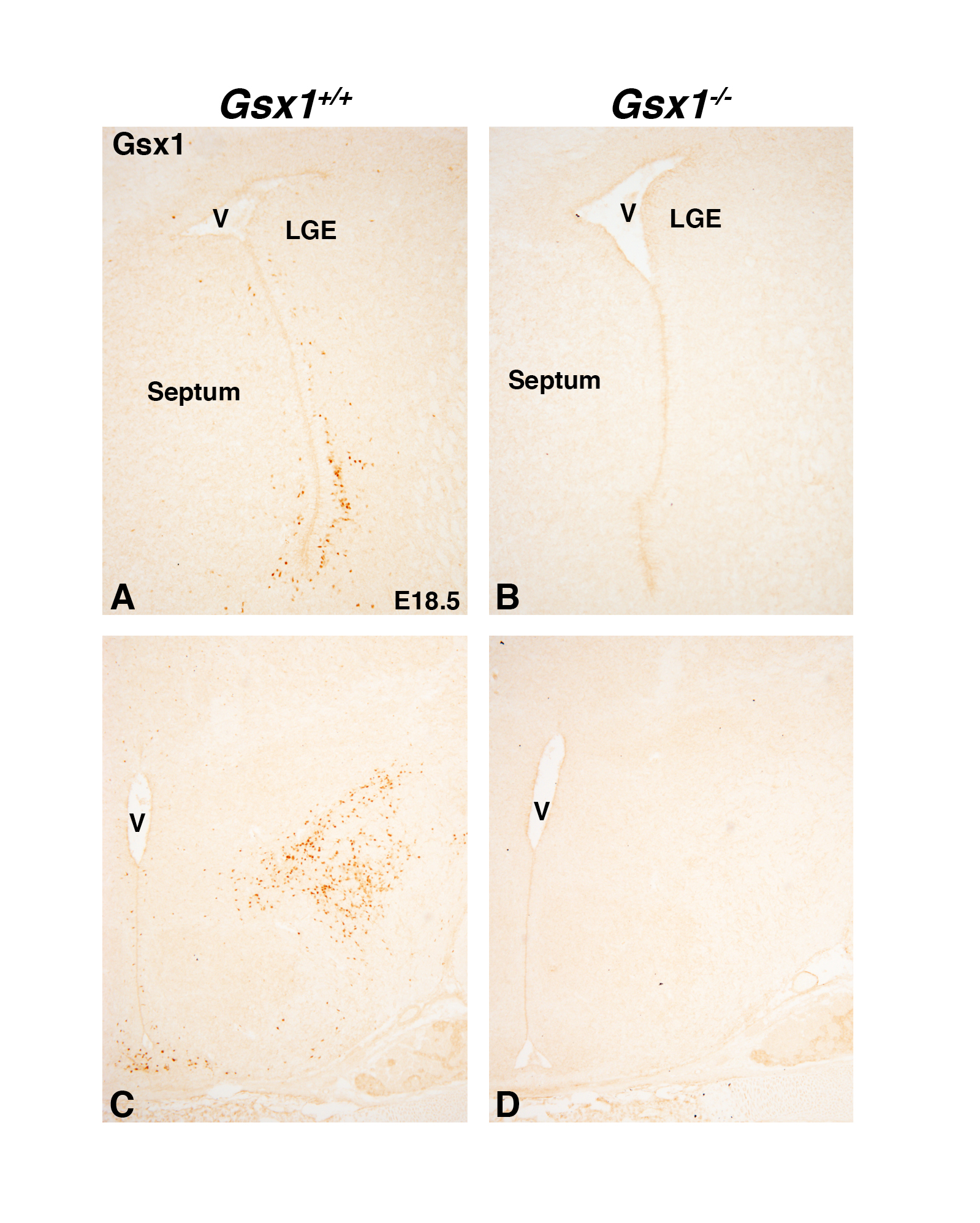

Supplement: Additional file 1: Figure S1. — Characterization of Gsx1 specific antibody. Immunostaining for Gsx1 in the telencephalon and diencephalon reveals positive cells in the ventral most LGE and developing hypothalamus (Gsx1 +/+) at E18.5 (A, C). No Gsx1 positive cells are detected in Gsx1 mutant (Gsx1 −/−) forebrain regions (B, D). (TIFF 9369 kb) [file 13064_2017_90_MOESM1_ESM.tif]
